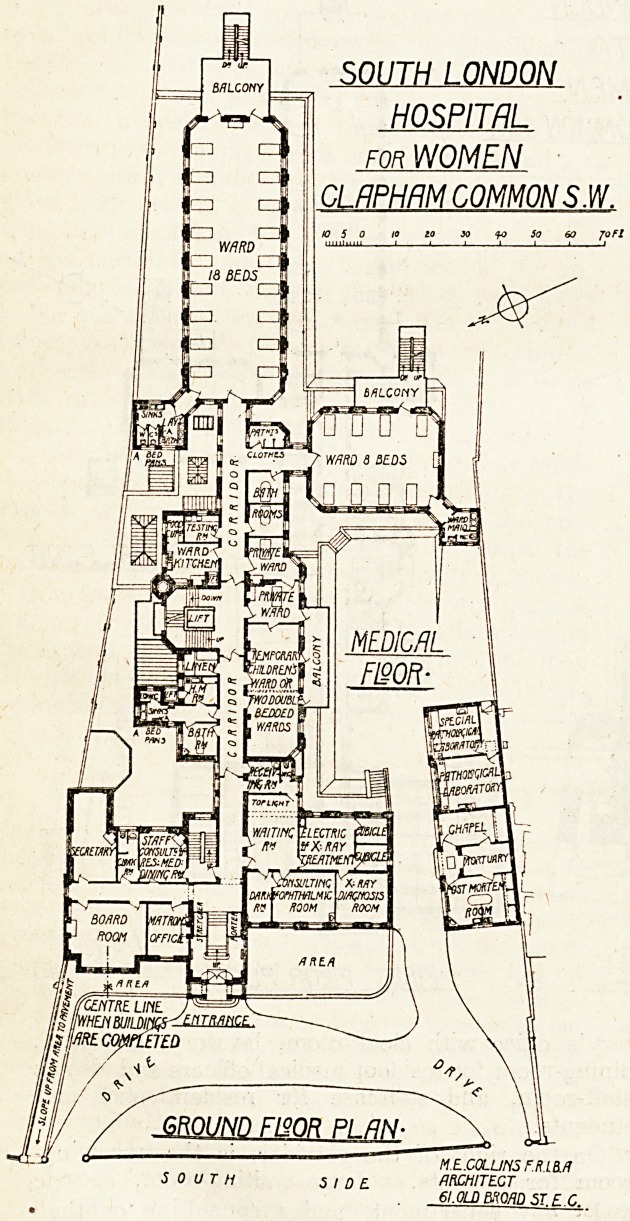# South London Hospital for Women

**Published:** 1916-07-08

**Authors:** 


### HOSPITAL ARCHITECTURE AND CONSTRUCTION.

### South London Hospital for Women.

This hospital has been established for the treatment of women by women physicians and surgeons, its objects being defined by the trust deed as "To afford to poor women and women of small means the opportunity of obtaining medical and surgical treatment by qualified women, and to promote the medical education of women, as also to educate and train medical students and nurses in all professional duties."

The new buildings, which were opened by Her Majesty the Queen on Tuesday last, are entirely devoted to in-patients, the present out-patient department being situated in Newington Causeway, S.E.

The site is on the south side of Clapham Common, and consists of two long narrow strips of land, the smaller of which is now occupied by a private house, the lease of which has still some years to run.

The new building, therefore, is of necessity long and narrow, and much ingenuity has been expended by the architect in contriving the necessary accommodation. The front of the building is set back between forty and fifty feet from the road. In the centre is the main entrance, in which ten steps arranged in two short flights lead up to the main corridor on the ground floor. To the left of the entrance are: matron's office, board-room, secretary's office with cloak-room, lavatory, and w.c., dining-room for resident medical officers and also as staff-room, and staircase for resident staff and students.

On the right of the corridor is the receiving-room for patients, a large waiting-room, electric and $x$-ray department, and a consulting ophthalmic-room with two dark-rooms. Swing doors across the corridors fixed centrally with the receiving-room, so that the two doors into the latter open one on each side of the swing doors, separate the wards and their offices.

Going eastward along the corridor on the right-hand side is a large room which is to be used temporarily as a children's ward, and when the hospital is extended will become two double-bedded wards; beyond this are two private wards, two bath-rooms, and the patients' clothes store. Between the bath-rooms and the clothes store is a short corridor leading to a ward for eight beds. The sanitary offices are placed in a wing projecting out from the south-west angle of the ward, and provided with a cut-off lobby. On the east side is a wide balcony, from which an escape staircase is arranged.

On the left side of the corridor is a bath-room with sanitary offices for the private wards, linen store, main staircase, in the well of which is placed the passenger-lift, ward kitchen, and small food store and testing-room.

At the extreme end of the corridor is a large ward for eighteen beds, with its sanitary offices projecting from the north-west angle, and a wide balcony with escape stairs at the extreme end.

The corners of the wards are cut off at an angle of 45 degrees, to correspond with the entrance to the sanitary offices, and in each angle is a window. This has the effect of making the ward symmetrical, but involves some loss of space. The wards each have an open fireplace, but for general warming reliance is placed on hot-water radiators.

On the first floor the left-hand part of the front building is occupied by rooms for the resident medical staff and students, with their bath-rooms and sanitary offices. On the right-hand side are nine private cubicles, separated by partitions 6 feet 6 inches high.

The remainder of this floor is similar to the floor below. On the third floor the front block contains two sisters' bedrooms, a sisters' sitting-room, nurses' sitting-room, "silence-room," with bath-rooms, etc., and the night nurses' bedrooms in a group separated by a door across the corridor. The rest of this floor, except the part over the eight-bed ward, contains nurses' bedrooms and the matron's sitting-room and bedroom.

Over the eight-bed ward is the operating department. This comprises a large top-lighted theatre, with surgeons' lavatory leading out of it; a smaller theatre for minor operations, a small sterilising-room, and the anæsthetic-room. The only way from the anæsthetic-room into the theatre is through the sterilising-room, so that a patient after operation must be taken back to the ward through the anæsthetic-room—a most unfortunate arrangement. The position of the surgeons' lavatory, which can only be reached by passing through the theatre, is also most unfortunate; the surgeon should only enter the theatre when fully equipped for operating. Outside the theatre is a room marked "retiring-room." What purpose this room is meant to serve is not evident, but it would have been more suitable for the surgeons' lavatory. No bath-room is provided.

A very complete isolation department is formed on the third floor over part of the centre block. It comprises two wards, each capable of holding two beds, with nurses' bedroom and ward kitchen and a bath-room which forms the entrance for nurses, and where they must change their clothing before going on duty. Their approach is by the staff stairs and across the flat roof, and not by the main staircase, which has no direct communication with the isolation department. The main stairs and lift both go up to this floor, and afford access to the flat roofs over the ward blocks.

In the basement are all the stores, kitchen offices, nurses' and servants' dining-rooms, servants' dormitories and sitting-room, and the boiler-house. The tradesmen's entrance, which is also used by the students and servants, is approached by an inclined road from the main road.

The pathological building is on the right of the main building, and contains mortuary, *post-mortem* room, chapel, and two pathological laboratories. It is a one-storey building, top-lighted from the north.

The architect is Mr. M. E. Collins, F.R.I.B.A.

## Figures and Tables

**Figure f1:**
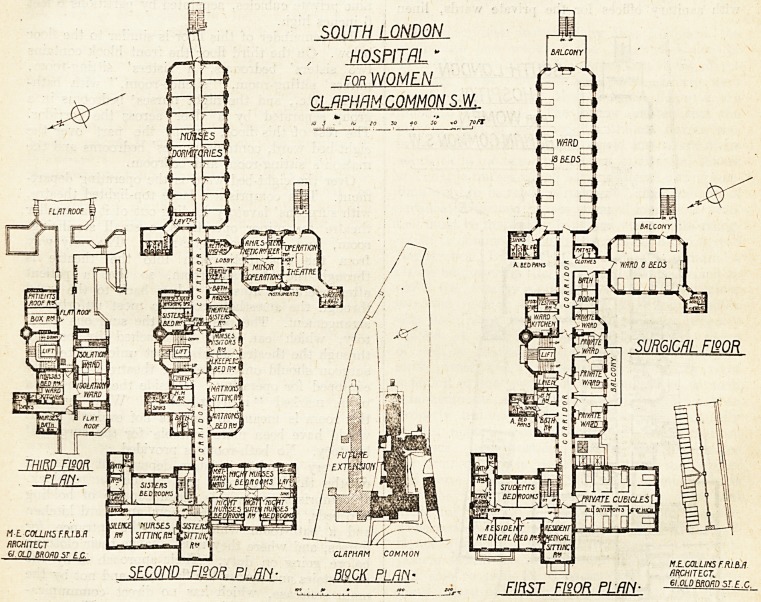


**Figure f2:**